# Comparison of 3 methods characterizing H_2_S exposure in water and wastewater management work

**DOI:** 10.1093/annweh/wxae043

**Published:** 2024-07-09

**Authors:** Åse Dalseth Austigard, Hans Thore Smedbold, Kristin von Hirsch Svendsen

**Affiliations:** Department of Industrial Economics and Technology Management, NTNU – Norwegian University of Science and Technology, PO Box 8900, Torgarden, N-7491 Trondheim, Norway; Trondheim Municipality, Working Environment Office, PO Box 2300, Torgarden, N-7004 Trondheim, Norway; Department of Occupational Medicine, St Olav University Hospital, PO Box 3250, Torgarden, N-7006 Trondheim, Norway; Department of Public Health and Nursing, Faculty of Medicine and Health Sciences, NTNU, PO Box 8900, Torgarden, N-7491 Trondheim, Norway; Department of Industrial Economics and Technology Management, NTNU – Norwegian University of Science and Technology, PO Box 8900, Torgarden, N-7491 Trondheim, Norway

**Keywords:** assessment strategies, exposure index, H_2_S, hydrogen sulfide, logbook, peak exposure, self-assessed data collection, wastewater

## Abstract

This study evaluates the effectiveness of self-assessed exposure (SAE) data collection for characterization of hydrogen sulfide (H_2_S) risks in water and wastewater management, challenging the adequacy of traditional random or campaign sampling strategies. We compared 3 datasets derived from distinct strategies: expert data with activity metadata (A), SAE without metadata (B), and SAE with logbook metadata (C). The findings reveal that standard practices of random sampling (dataset A) fail to capture the sporadic nature of H_2_S exposure. Instead, SAE methods enhanced by logbook metadata and supported by reliable detection and calibration infrastructure (datasets B and C) are more effective. When assessing risk, particularly peak exposure risks, it is crucial to adopt measures that capture exposure variability, such as the range and standard deviations. This finer assessment is vital where high H_2_S peaks occur in confined spaces. Risk assessment should incorporate indices that account for peak exposure, utilizing variability measures like range and standard or geometric standard deviation to reflect the actual risk more accurately. For large datasets, a histogram is just as useful as statistical measures. This approach has revealed that not only wastewater workers but also water distribution network workers, can face unexpectedly high H_2_S levels when accessing confined underground spaces. Our research underscores the need for continuous monitoring with personal electrochemical gas detector alarm systems, particularly in environments with variable and potentially hazardous exposure levels.

**What’s Important About This Paper?**

This study demonstrates that highly variable exposures with acute effects, such as occur with hydrogen sulfide in the wastewater industry, will be poorly described using targeted or randomized shift-duration sampling. Data-logging electrochemical sensors used to warn workers of imminent hazard provide more robust exposure characterization.

## Introduction

Different kinds of exposure assessment and sampling strategies have been proposed over the years. Historically, exposure characterization has predominantly relied on time weighted average (TWA) exposure values, given the emphasis on cumulative dose and long-term impacts, as well as the feasibility of obtaining such measurements (Borak and Brosseau 2015; Wheeler et al. 2015). Workplace exposure measurement standard NS-EN482 (Standard Norge 2021) was established on this premise. Furthermore, the selection of representative measurement days is integral to this approach, acknowledging the impracticality of daily expert exposure measurement. Standard EN689 (CEN 2019) is one of the standards that provide statistical guidance for evaluating these assessment strategies and measurements. However, it is solely—as are most other similar standards—intended for assessing the mean in relation to the 8-h occupational exposure limit (OEL) or the short-term exposure limit (STEL) (typically 15 min).

Other exposure assessment strategies have been proposed. Olsen (1994) presented Logbook as a method of collecting metadata on process-based exposure measurements. Descatha et al. (2022) present Hoar (1983) as the first to describe the job exposure matrix (JEM) as a way to assign exposure to specific jobs within a group of workers. Some kind of logbook method is essential to collect the parameters needed to develop task exposure models. The method has been frequently used to describe exposure (Peretz et al. 2002; Dopart and Friesen 2017). It simplifies the underlying exposure profile to a set of numbers describing the exposure of interest, often the TWA. The range or confidence limits of the TWA (or other mean values), the peak values, or the full exposure profiles, have seldom been used. Usually, exposure measurements are planned and performed by occupational hygienists. However, Pettersson-Strömbäck et al. (2008) have examined the quality of self-assessed exposure (SAE) data, compared to expert collected data. They found that SAE measurements give more valid estimates compared to expert measurements, but that the necessary follow-up requires expert advice and formal organization.

The use of TWA as a regulatory term is based on Haber’s Law (Haber 1924), which states that dose to effect is a constant relationship between concentration and time. Not all chemicals follow this, and hydrogen sulfide (H_2_S) is one of them (Guidotti 1996). Chronic effects may result from both long-term exposure and by short exceedances of a trigger level, as seen for H_2_S (Svendsen 2001; OSHA 2023). H_2_S exhibits both chronic and multiple acute effect levels, and relying solely on TWA evaluation could underestimate the associated risks (Smith 2001; Virji and Kurth 2021). Relying only on peak levels could overestimate or fail to recognize chronic effects (Flegal et al. 1991). Consequently, an H_2_S-index that incorporates elements of TWA data, ceiling value (CV) exceedance, and the number and magnitude of peaks was introduced (Austigard et al. 2018) allowing for an integrated representation of the exposure risks. This approach was developed in response to the inadequacy of low 8 h TWA to explain possible health effects observed from H_2_S exposure, and to provide employees and employers with a single term for risk evaluation rather than multiple levels (Austigard et al. 2018). Additionally, an algorithm has been proposed to automate this calculation process (Austigard and Smedbold 2022).

Exposure to certain gasses, such as H_2_S, exhibits high acute or chronic potential that makes continuous monitoring essential. Personal electrochemical gas detector alarms with data logging (alarm equipment) that register exposure levels over time, are increasingly utilized in water and wastewater management operations, but they are not yet mandatory in Norway. Trondheim municipality, however, has employed such sensors since 2013. Recent research has demonstrated that the data from these devices can serve not only for documentation of alarms but also as exposure documentation (Austigard et al. 2023).

The need for precise exposure assessments has been highlighted in several papers (Kromhout 1994; Kriebel et al. 2007; Smith and Kriebel 2010). Prior research on task and group descriptions (Austigard 2023) has indicated that water distribution network work warrants further scrutiny due to previously observed unexpected high peaks. If these peaks are attributable to inadequate exposure comprehension for specific tasks, it could imply the absence of crucial exposure information. Additionally, this may suggest that a control group of water workers are not unexposed to H_2_S, potentially affecting risk ratio findings in other groups within an epidemiological context (Stewart et al. 2000; Blair et al. 2007).

The aim of this study was to compare 3 sampling strategies used to characterize water distribution workers and wastewater workers exposed to H_2_S. As part of the comparison, a third dataset (C) is presented and the use of an exposure index for H_2_S is explored.

## Method

**Fig. 1.**
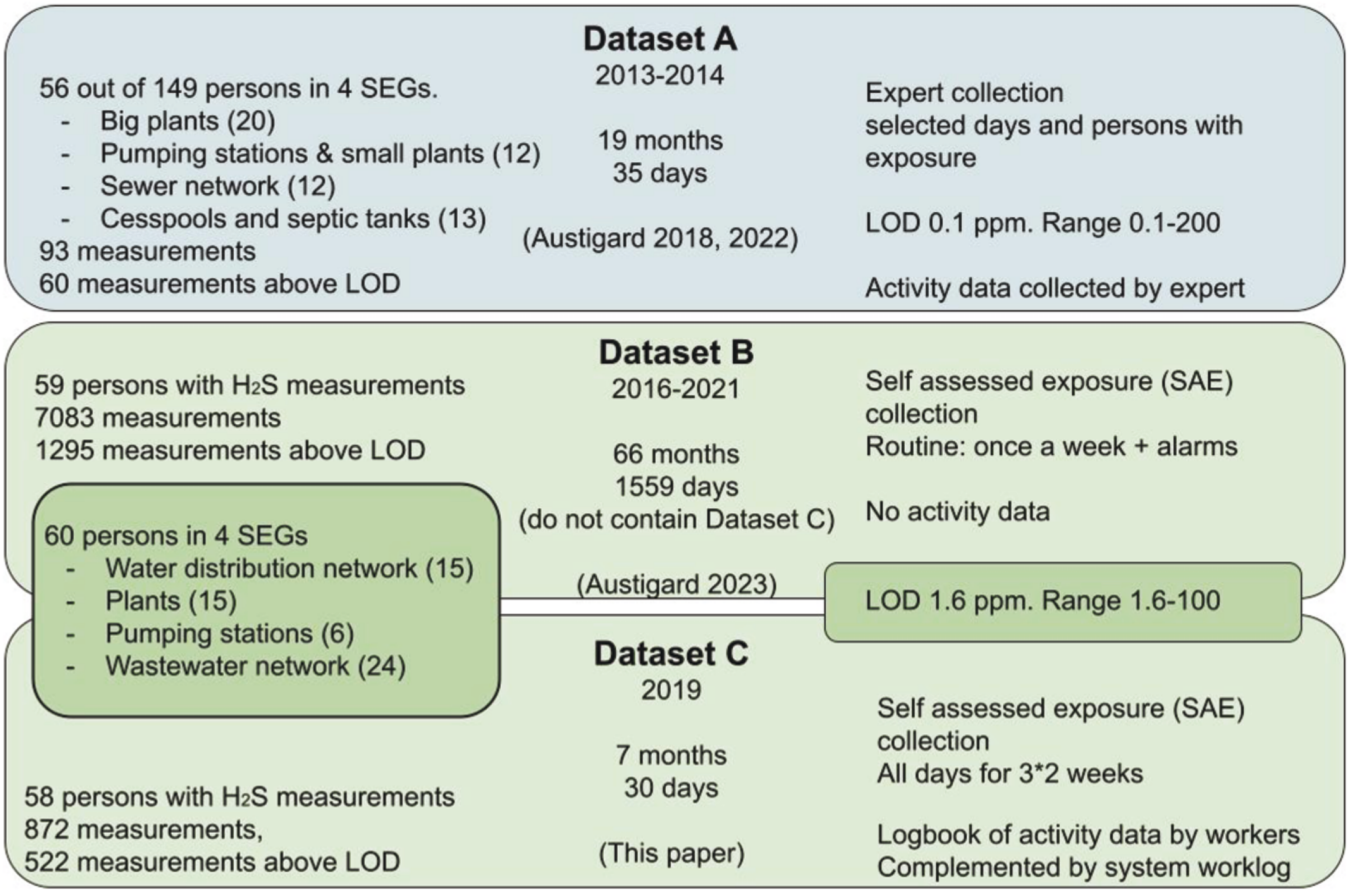
Illustration of the specifications, differences, and connections among the 3 datasets and publications. The boxes overlapping datasets B and C illustrate that the same people and equipment are involved. “Days” refers to days with measurements, and “months” refer to study duration. H_2_S measurements in dataset A are from a larger dataset which includes endotoxin and bioaerosol exposure measurements in a total study group of 149 persons. No measurement was duplicated between dataset B and dataset C. One person was in 2 groups in dataset A.

Below are short summaries of the methods of datasets A (Austigard et al. 2018; Austigard and Smedbold 2022) and B (Austigard et al. 2023), and a more detailed summary of dataset C, with their distinctions, characteristics, and methodology for comparing the results. The studies are summarized in Fig. 1. There is no overlap of exposure data between the datasets.

### The three datasets

For *Dataset A* the collection was made in the Trøndelag region and the Oslo area from 2013 to 2014. The assessment strategy was expert collection on measurement equipment OdaLog L2/LL (range 0.1 to 200 ppm, Thermo Fisher Scientific, Australia), Dräger Pac 7000, and Dräger x-am 5000 (range 0.1 to 100 ppm, Drägerwerk AG & Co KGaA, Germany). The dataset contains 93 measurements of H_2_S for 56 persons out of a total study group of 149. Measurements cover 35 workdays, over a study duration of 19 months. The resulting graphs were assessed manually to count peaks and durations in different concentration intervals to calculate the index value for H_2_S [further detail in Austigard et al. (2018)]. Workers influenced the number of H_2_S measurements in dataset A by sometimes stating that they would not be doing anything H_2_S-related that day, while the plan was to make H_2_S measurements in parallel with the other measurements (endotoxins and bioaerosols) of the study (Heldal et al. 2019).

*For both datasets B and C*, the datasets were collected in Trondheim, the 3rd largest municipality in Norway with some 212 000 inhabitants (SSB 2022). It is part of the Trøndelag region. All 65 employees in the water and wastewater department of Trondheim municipality who were provided with alarm equipment for measuring H_2_S exposure were invited to participate in the study. A total of 60 individuals chose to participate. They constituted 4 similar exposed groups (SEG), with 24 working in the wastewater network group (which includes the emptying of septic/cesspools), 15 in sewage treatment plants, 6 with pumping stations, and 15 with the water distribution network. Personnel in the last group enter manholes in the water distribution system. The 5 people who did not participate mainly performed office work.

The study was approved by the employer, and a consent form was developed in collaboration with the union representative for water and wastewater workers and the privacy representative of Trondheim municipality. The research proposal was presented to the workers as the consent form was disseminated. Participants were included in the study through an opt-in strategy. Six individuals opted not to participate, due to their main engagement in office work.

Measurements consisted of SAE-collected alarm registrations and measurement logs from BW MicroClipX3 (Honeywell International Inc., USA) personal electrochemical gas detector alarms with data logging (alarm equipment) with a measurement range of 1.6 to 100 ppm H_2_S. The interval between data points was 15 s. Storage capacity at this rate was 16 h of wraparound (Honeywell 2020). Alarm registration was stored in a separate wraparound slot, so that the maximum at alarms could happen to be extracted without the corresponding measurement log, giving a single datapoint. The storage unit could skip transfers of all zero data intervals at transfer to a spreadsheet. Distributed docking stations were available, and ensured bump check and data transfer, as well as calibration if due (180 days). Instruments were mostly placed in the breathing zone, but some were worn at the waist.

*Specific to dataset B*: the routine of docking said at least once a week and after alarms. The dataset covers 1559 days over a study duration of 5.5 yr from 2016 to 2021. It contains nearly 7100 days with registration for 59 persons out of 60 enrolled in the study. The SARS-COV-19 pandemic negatively affected the collection of data. Further detail in Austigard et al. (2023).

*Specific to dataset C*: the exposure assessment strategy was to measure every day for 3 × 2 wk (February/March, June, and September) and maintain a register of activity information. During collection of dataset C the workers should dock their equipment every day. Data was divided into SEGs. The dataset covers 60 days over a study duration of 7 mo in 2019. It contains 872 days with measurements for 58 persons out of 60 enrolled. Temperature, wind, and precipitation data were collected.

A logbook form (Translated version in Supplementary I) was discussed with the workers, and adjusted with work categories accordingly. In the first 2-wk period, the logbook was given in paper form. For the next 2 periods the logbook was distributed electronically as a Google form. Workers were encouraged to answer throughout the day as work progressed. Days with no information from sensor or logbook were controlled against the work log system and divided into “real zero exposure” (caused by for example sick leave, holidays, education days, union work, and scheduled compensation days for on-call work), and “missing data”. For more details see Supplementary II.

The logbook activity information was converted to variables of real zero exposure, workplace, flushing activity, and number of exposed tasks. These were combined with the aggregated workday exposure information from the alarm equipment. The logbooks had to be manually assessed, as it was obvious from the data that “duration” was often registered as “end time” and therefore affected task time. Measurement days at weekends and holidays with only zero exposure were removed from the data unless there was also activity registration for the person. Entries with information for multiple days were separated to their correct dates. A few people had marked that they had used other alarm equipment. Data from these measurements were added to the dataset if they had provided sufficient information to find the correct data.

The data were aggregated to one log per person and day by a previously published algorithm (Austigard 2023). A visual presentation of the data with 3 risk measures of H_2_S in the same graph (index value, TWA, and exceedance of CV) is provided. The index was calculated as:


$$\begin{array}{lll} Index=\; & H_{2}S_{01}*0.1+H_{2}S_{Duration01}*0.1+H_{2}S_{1}+\; & H_{2}S_{5}*5+H_{2}S_{Duration5}*5+H_{2}S_{10}*10+H_{2}S_{max} \end{array}$$

where

H_2_S_01_ is number of peaks in the interval 0.1 to 1.0 ppm,

H_2_S_1_ is number of peaks in the interval 1.1 to 5.0 ppm,

H_2_S_5_ is number of peaks in the interval 5.1 to 10.0 ppm,

H_2_S_10_ is number of peaks in the above 10.0 ppm,

H_2_S_Duration01_ is duration in minutes between 0.1 and 5.0 ppm,

H_2_S_Duration5_ is duration in minutes above 5.0 ppm,

H_2_S_max_ is the maximum H_2_S level detected.

### Comparison of datasets

In the meta-analysis of the 3 studies, the outcome was compared descriptively by frequencies in exposure intervals, and by comparing results from ANOVA mixed model analysis. Whether a parameter improves the model or not is evaluated by comparing the models’ −2-logLikelihood values. If the value decreases, the model gives a better fit to the data. The model assumes variance components, meaning estimating the contribution of each random effect to the variance of the dependent variable (IBM 2021). For comparison of results across the 3 datasets, the ANOVA analysis was based on SEG and season, as all datasets contained these parameters. Wastewater network workers and cesspool emptiers were 2 different groups in dataset A, but combined in dataset C. In dataset A, no water distribution network workers had H_2_S measurements, as they were assumed to have zero exposure. For the comparison, results were calculated based on values below LOD being imputed in the same way as the original presentation (Austigard 2018), with “lowest index value”/sqrt(2). In dataset A this gave 0.283, in datasets B and C it gave 1.927.

To evaluate the robustness of dataset C, positive measurements with activity data were divided into 2 by iterating random draws of datapoints, until approximately 50% (±2.5 %) of data points were present in each part and at the same time 50% (±3.5 %) in each SEG. The ANOVA mixed model analysis was run on the 2 parts separately, and the models were compared to each other and to the full dataset.

## Results

Dataset C and the results from the comparison of the 3 datasets is presented below.

### Dataset C

In total, 1807 workdays were involved, including 7 days identified as on-call work on weekends and a public holiday. H_2_S was detected on 522 (29 %) of the workdays, equaling 60% of the workdays with measurement. Peak values exceeding CV were found on 118 workdays (7% of total, 14% of measured). Real zero days were rarely covered by the measurements, even though the participants were asked to use the equipment every workday. Real zero days account for 23% of the total number of workdays. Context information was present for 81% of the workdays. Data was missing for 11% of workdays. Further details are given in Supplementary Material.

**Fig. 2.**
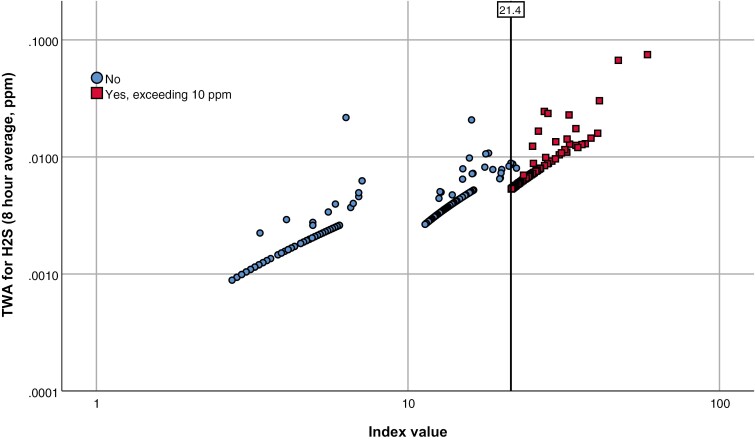
Index to TWA for H_2_S, separated into workdays with [*N* = 118 (squares)] and without [*N* = 404 (circles)] peaks exceeding 10 ppm. The bar at index value 21.4 symbolizes the lowest possible index value of measurement when exceeding 10 ppm during a workday. The 3 arches forming the bottom of the figure are due to the index algorithm multiplication at different levels of H_2_S. This is most visible when formed by measurements with a single data point above LOD. Regression values (coefficient of determination) for linear fit lines are *R*^2^_total_ = 0.819, *R*^2^_No_ = 0.758, *R*^2^_yes, exceeding CV_ = 0.813. The axes are in logarithmic scale. The “Y” axis is in decimal scale in scientific notation. “1.E−1” equals 0.1 ppm. This is 1/20 of the OEL at 5 ppm.

All calculated TWA values were below the minimum TWA level at 0.1 ppm which can be shown on the instrument itself. One measurement had the highest value on all 3 parameters: TWA (0.07 ppm), peak (29 ppm), and index value (59), and belonged to a water distribution network worker. A selection containing the highest 20 values on each parameter are shown in the Supplementary Material. Figure 2 visualizes the results by plotting the index value against TWA, and by distinguishing between values exceeding CV (red circles) or not (blue circles). The figure shows that when evaluated only by TWA value, risk is considered satisfactory, but CV evaluation shows multiple exceedances, indicating that the risk related to this type of work is still significant.

**Fig. 3.**
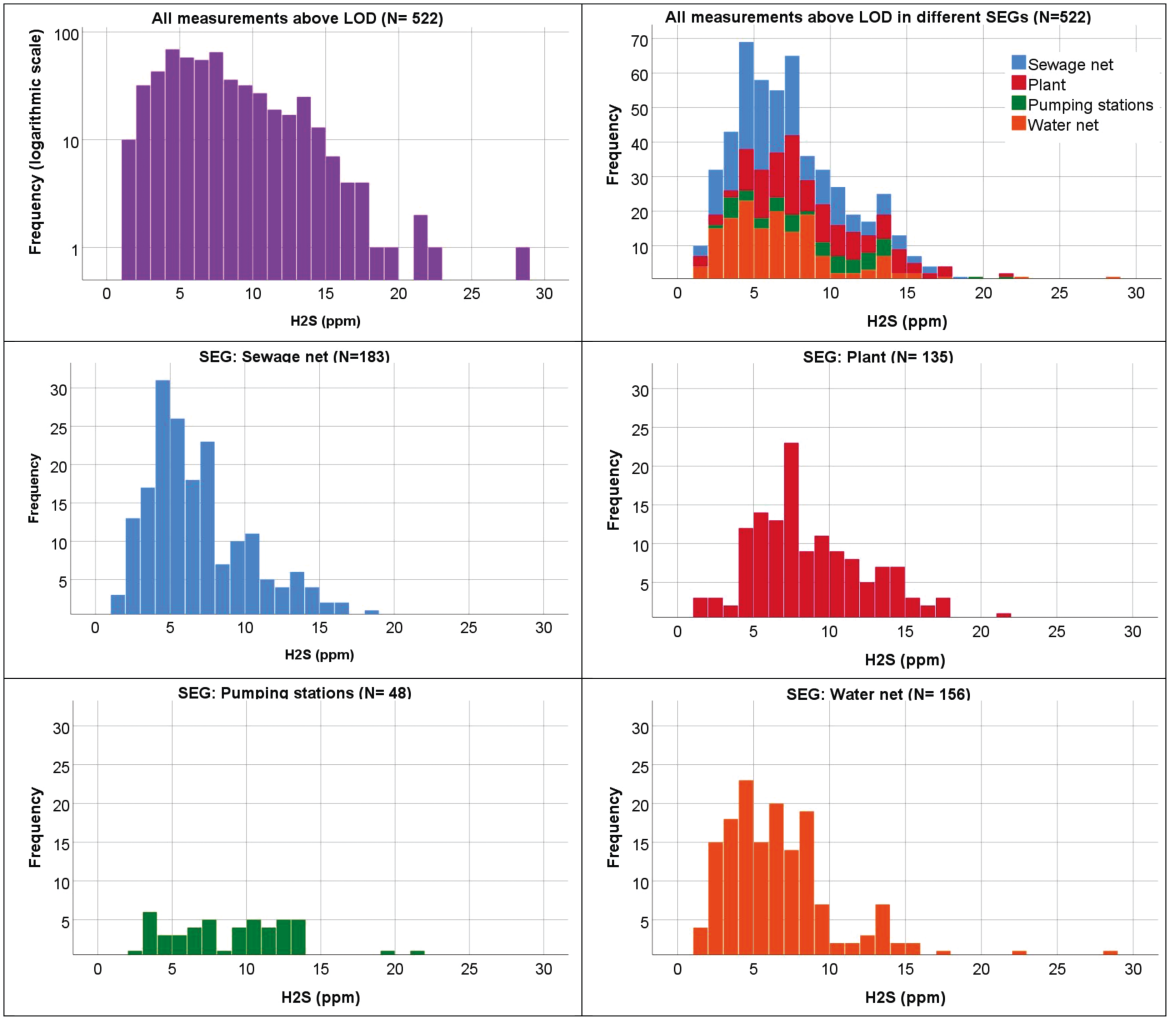
Distribution of maximum H_2_S level in measurements for a workday with recording every 15 s, illustrating how common it was to reach different maximum levels of H_2_S in the water distribution network and wastewater-related work, and to exceed the ceiling value of 10 ppm. Total *N* = 522. Upper row: all measurements above LOD (mean 7.5 ppm, SD 3.9 ppm). Upper left: logarithmic “Y” axis. Upper right: stacked total on linear “Y” axis. Middle and bottom row separate for the SEGs. Linear axis in the same scale. Not corrected for group size.

The histograms shown in Fig. 3 of maximum H_2_S level on workdays give a log-normal impression, with a mode between 5 and 8 ppm. Most workday measurements have only one positive recorded value, those with most have 15, corresponding to 4 min a day. There was no correspondence between the number of positive values in a measurement and exceeding the CV.

Water distribution network workers had many incidents of measured exposure that could not be explained by their categorization of work tasks, dominated by “driving” and “unexposed water network tasks”. This also accounts for the registrations exceeding the CV. Activity data showed that they reported entering manholes 11 times more often than wastewater collection system workers (34 versus 3 during these 6 wk). Corrected for group sizes, the entering of manholes happened 18 times more often for water distribution network workers, than for wastewater collection network workers. For the water distribution network, 4 of the manhole entries were not covered by measurements, 7 had measurements where all registrations were below LOD, 22 had measurements with registrations between LOD and CV, and one above the CV. Wastewater collection network workers have one observation in each interval. The other 2 SEGs do not report any entering of manholes.

### Comparison of the 3 studies

Table 1 provides descriptive numbers for the 3 datasets and complements the information in Fig. 1.

**Table 1.** The number of H_2_S observations in the 3 datasets; expert collection (A), self-assessed exposure (SAE) collection by routine once per week + alarms (B), and SAE collection every day in the campaign period (C). The total number of “study workdays” in each study was calculated as the “days” (with measurement) multiplied by the number of persons in the study, while “study duration workdays” was calculated from the number of months and 220 workdays a year per person. *N* is the number of workdays with measurement. The ceiling value (CV) is 10 parts per million (ppm).

	Dataset A (*N* = 93)	Dataset B (*N* = 7083)	Dataset C (*N* = 872)
Number of measured workdays with recordings above 1.6 ppm (% of *N*)	25 (27%)	1295 (18%)	522 (60%)
Number of workdays with recordings exceeding CV (% of *N*)	8 (9%)	424 (6%)	118 (14%)
Workdays exceeding CV in % of above 1.6 ppm	32%	33 %	23%
Number of workdays with recordings above 100 ppm	1	3	0
Number of study duration workdays (% measured)	51 902 (0.2 %)	72 600 (10 %)	7700 (11 %)
Number of study workdays (% measured)	5215 (2 %)	93 540 (8 %)	1807 (48 %)

In dataset C, the partition into 3 periods during the study covered 4 seasons according to the partition in dataset A. A very small improvement was seen in the −2-loglikelihood value when divided into 4 seasons rather than 3 test durations. When looking at measurements with activity data, the variables SEG and season contribute significantly to improving the model. Flushing was used as a category by only 2 SEGs. Eighty three percent of workday measurements were not registered with any flushing. Flushing was not significant for dataset C.

Table 2 shows a comparison of calculated AM of index values with 95 % confidence intervals for some situations based on the parameters from the ANOVA mixed models. The two models based on random draw of data, have some parameters outside the confidence limits when including all data. The resulting calculations of AM are affected, but rest upon more than one parameter, and therefore become within limits.

**Table 2.** Comparable calculations of arithmetic mean (AM) of H_2_S index value from ANOVA mixed model based on datasets A, B, and C with values imputed below LOD as “lowest index value/sqrt2.” Values are given with a 95% confidence interval in brackets. Index values are without units. The values 23 confirmed real zero are not imputed in dataset C. Index values have no unit.

	Dataset A (*N* = 93)	Dataset B (*N* = 7083)	Dataset C (*N* = 849)
Pumping stations in autumn	6 (1 to 56)	4 (3 to 5)	8 (5 to 11)
Plant in summer	16 (0 to 361)	4 (3 to 5)	8 (4 to 19)
Sewage network and cesspools in winter	23 (8 to 68)	4 (4 to 4)	7 (6 to 9)
Plant in spring	1 (0 to 45)	4 (3 to 5)	11 (5 to 24)
Plant in winter	8 (0 to 64)	4 (3 to 5)	11 (6 to 21)
Plant in autumn	2 (0 to 53)	4 (3 to 5)	11 (5 to 26)
Pumping stations in summer	47 (6 to 382)	4 (3 to 5)	6 (4 to 8)
Water network in autumn	4 (0 to 119)	4 (3 to 5)	13 (7 to 29)

**Fig. 4.**
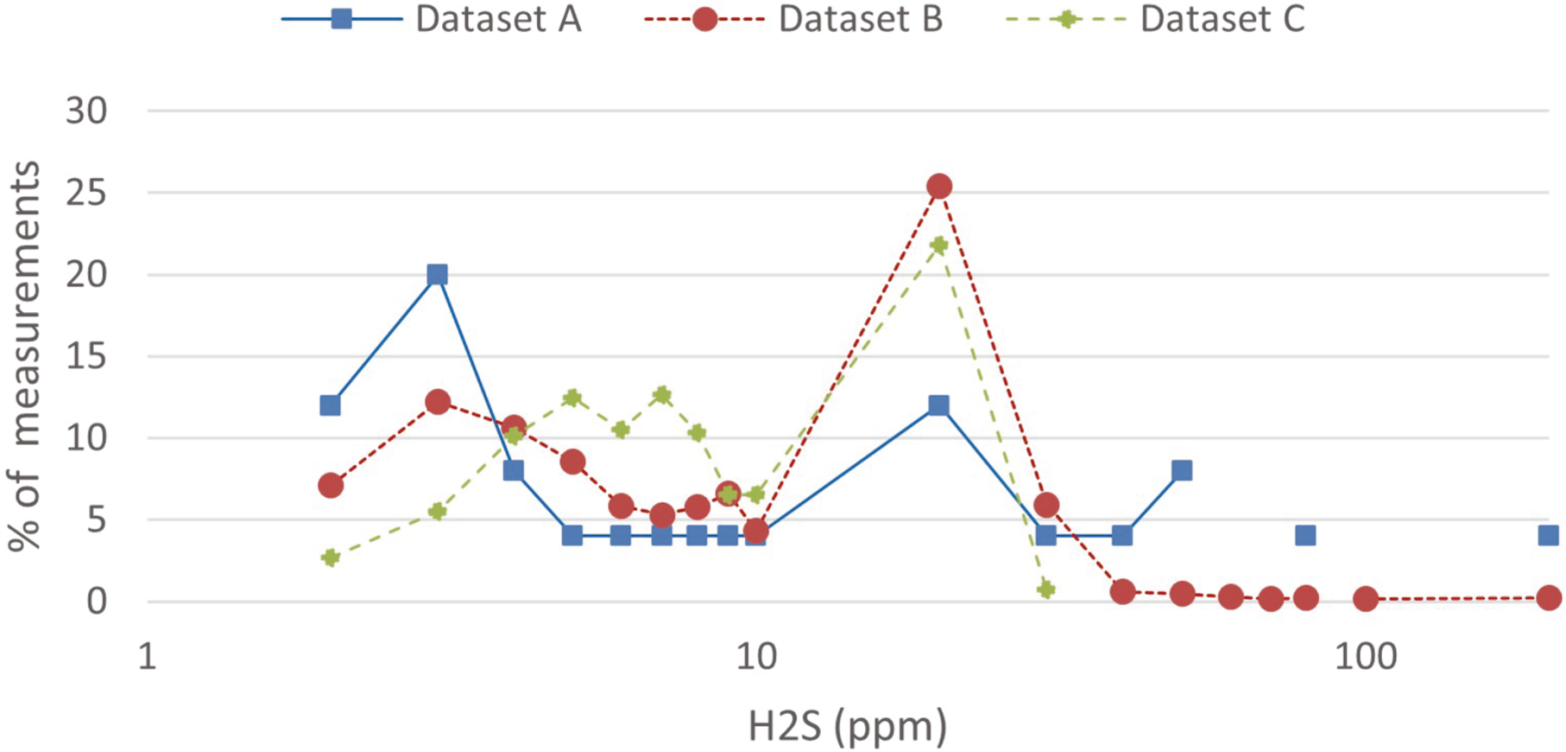
Distribution of maximum H_2_S value per workday in ppm in measurement above 1.6 ppm, which was the mutual measurement area for the datasets. Differences in equipment and collection strategy affects the fractions. Values are binned as “<*x*<=”. Bin size is made by 1/10 of upper 10-integer, so that in the interval 2.1 to 10.0, the bin size is 1. From 10.1 to 100 it is 10. The bin endpoint 10.0, was chosen as this is the CV that should not be exceeded. Values between 1.7 and 2.0 are the first bin shown, as LOD for B and C sampling was 1.6 ppm. Values above 100 ppm are in one bin. In datasets B and C this equals overload of alarm equipment. Lines are broken when no fraction is present in the bin.

The distribution of peak values from the 3 studies is shown for values above 1.6 ppm in Fig. 4. Concentration levels are binned by evaluating “bin min < × <= bin max.” Values from 1.7 to 2.0 are the first bin shown, as LOD for B and C sampling was 1.6. All 3 methods reach 90% of accumulated positive measurements just before 20 ppm. Values above 100 ppm are shown as one bin.

## Discussions

The different strategies give different results. How this affects the ability to characterize the exposure and assess and handle the related risk is discussed.

### Comparison of the 3 studies

Three different assessment and sampling strategies are compared. The data shows that the strategy for dataset B gives the best picture of the exposure profile. However, the strategy for dataset C provides the opportunity to find earlier unrecognized exposed tasks because of the metadata on activity. Notwithstanding, due to the random nature of the exposure it cannot be expected to get the highest peaks, as extraordinary tasks can easily be missed. This also accounts for dataset A, but A provides more opportunity to choose days with exposure. The strategies behind datasets A and C demand extra effort from both workers and experts, making the strategies time consuming.

The difference in sampling strategy contributes to the differences in detected exposure levels, as shown in Fig. 4. Low TWA levels are expected, as exposure largely consists of peaks. Fractions of days with a maximum level between 3 and 10 ppm are lower in dataset A than in datasets B and C, as measurements in A focus on highly exposed tasks, while those in B also have routine collection, and those in C focus on full coverage of the period without any task preference. This also results in higher proportions of values above 20 ppm in A, than in the other 2 studies.

The median level of H_2_S in dataset A was below LOD in datasets B and C, which is an effect of the measurement equipment in dataset A having a lower LOD than in datasets B and C. The effect on the index values is, however, smaller. This is due to the calculation method of the index, as levels below 1 ppm have minor influence on the calculation (Austigard et al. 2018).

With a daily sampling strategy (dataset C) a much higher proportion of exposed workdays was found than in earlier published data (A and B). Fractions of days measured that are above LOD at 1.6 ppm have tripled from dataset B to dataset C, from 18% to 60%, and doubled from the 27% in dataset A. The fraction of exceedances of CV in total measurements has not increased as much (from 9% and 6%, to 14%), as shown in Table 1. The fraction of days above LOD that exceeds CV has declined from 33% in dataset B to 23% in dataset C. These 3 comparisons suggest more exposed days are found with a daily sampling strategy as in C, but relatively more below CV than above. This does not give more covered work time during the observation period (months) than routine collection, like in B, but it gives a more complete picture of the exposure on the measured days. This difference in the fraction of days with detected exposure between the datasets is an indication that workers overestimate their skill to predict when exposure to H_2_S occurs, and is also a reminder to occupational hygiene professionals of the same. This is in agreement with the findings of Logan et al. (2009), that qualitative judgment (i.e. without any measurement data) has an accuracy around that of random chance. With some measurement data and statistical training, accuracy was increased from 43% to 63% (Logan et al. 2009). This underestimation of exposure also accounts for exceeding CV, which makes alarm equipment important for health and safety. Numbers are found in Table 1 and in the Supplementary Material.

The detection limit suppresses the number of detected values per day. This increases the number of workdays with only “nondetects”. However, the data contains a large number of real zero days. Lavoue and Burstyn (2021) presented a method for calculating the proportion between real zero and LOD when this is not known. They showed that if real zero is not acknowledged, a large proportion of censored data where a substantial part is true zeros, can bias the risk assessment toward noncompliance. In occupational hygiene statistics, when comparing the results to a TWA, this could be a problem. It has been argued that the 8-h TWA and its 95th percentile do not reflect the risk in wastewater work (Austigard et al. 2018, 2023). Even so, the true zero analysis is of interest when calculating potential occupational health effects. The plan was to also evaluate the measurement results through ANOVA mixed model results, as originally made in dataset A, but the fraction of measurements below LOD is 30% to 80% of the datasets. This makes imputation with a fixed value, not a valid method to use. Excluding values below LOD is also inappropriate for calculating the parameters. Neither will a calculated mean of the index represent the health risk well, as it is the high-end levels that oppress the risk, not the mean. No statistical distribution method bases will represent the high-end exposure of datasets with so large proportion of zero values. These challenges are illustrated by the results in Table 2. For some example calculations see the Supplementary Material.

The expert strategy of collecting dataset A means measuring on a few individuals and on a few days over a period of time under the assumption that the exposure does not vary too much, and results are valid for the other days and other workers. Using alarm equipment and routinely collecting the data might demand an effort from the worker to remember to download data. By automated dumping of data when running bump checks, there is less effort for the worker, but activity data are not collected. This is the situation in dataset B. In total 8% of study workdays are covered. This is 4 times more than the expert collection for dataset A, but 14% of the intensified collection in dataset C. Both datasets with an SAE strategy cover approximately 10% of the total number of workdays during the study period, but their distribution is different: dataset B involves a few persons and almost each day. Dataset C involves a few days (full selected weeks) with almost every person. The strategy of dataset A covered 0.2% of study duration workdays.

Cross-sensitivity is not likely to explain the measured values of H_2_S among water distribution network workers. Documentation from Honeywell (2017, 2019) states the cross-sensitivity of the H_2_S sensor in the equipment to CO is <0.6%, giving an additional reading of 0.1 ppm H_2_S at 15 ppm CO. If linear, this means that CO level at 200 ppm (high-level alarm, IDLH) will not result in a reading of H_2_S above LOD. This complies with findings of low correlation between sensors, presented in a poster at the 10th Air Monitoring Conference (AIRMON10) (Austigard et Al. 2022b).

The use of alarm equipment requires addressing logging intervals. Alarms should come as quickly as possible when passing relevant levels. The *T*_90_ time of alarm instrumentation, whether personal or not, should therefore be short. When logging the data, the logging interval should be just above the *T*_90_. These instruments seem to use instant value at storage time, rather than a mean over the logging interval. Such choices in equipment make them easier and cheaper to produce. The 15-s logging intervals give a good representation of the exposure of H_2_S, and *T*_90_ at this level gives a quick alarm response when needed. It was shown that autocorrelation in the equipment used is low and that it is dominated by the correlation between zero values (Austigard et al. 2023).

Tasks that are rarely done, but have extremely high exposure, need to be planned into a measurement regime to secure the exposure data. This was not done for datasets A and C. Workers tend to promote the measuring of work they think of as highly exposed. Dataset B collects all such high exposure as long as data is transferred according to the routine. The strategy for dataset C will give a better representation of the most common exposure situations below the 10 ppm CV. These will be underrepresented in dataset A as workers influence which situations are measured and in dataset B as the focus is on alarms. Figure 4 illustrates this. The unpredictability of H_2_S exposure should be expected to amplify this effect.

Collecting alarm data adds value to the use of personal protective equipment (PPE). It gives opportunities for prevention by finding highly exposed situations, and documents such exposure. Quality of data declines if there is no feedback from the workers on the data collected. Systematic collection of alarm data also reduces misclassification of work situations as unexposed. Exposure assessment is an art where it is necessary to know the workplace in question. Even then, there might be surprise exposures. The effects of the exposure in question provide implications for assessment strategy. For chronic effects, it is common to assume TWA as a relevant measure, while acute effects need peak measures as shown.

Work on the water distribution network was not measured for H_2_S during collection of dataset A, as both experts and workers assumed it to be unexposed to H_2_S. Due to the collection strategy of measurements and activity data in dataset C, it was seen that some of the work categorized as “waternet work without sewage present” must nevertheless be exposed work. One explanation could be that manholes in the water distribution network are connected to the sewer through drainage. If so, H_2_S and methane are bound to be present to some degree.

Previously it was indicated that water distribution network workers might also be exposed to high levels of H_2_S (Austigard et al. 2023). This is also seen in dataset C. This time, the exposure stood out in frequency and level because all the other SEGs were lower in maximum exposure than in the other datasets (Table 1 and Fig. 3). It appears that water distribution network workers are much more likely to be exposed to H_2_S than expected based on their own description of the work.

### Index evaluation

Notation of exceeding the CV does not distinguish between peaks of different heights, just between exceeding or not. Like the use of TWA when dominated by peaks, this is problematic when there are many different effect levels. Even at levels when the worker does not recognize an acute effect from H_2_S, an impact on the body can be present (Bhambhani and Singh 1991; Bates et al. 2002). The index facilitates a measure for parts of this problem by including the maximum level present and different weights to time at different intervals of exposure. A further division in intervals of peak level in the high-end could be useful. This particularly applies if the profile shows higher proportions in the high-end exposure interval than found in these studies, and if the IDLH level is exceeded.

The use of data in a mixed model analysis depends on metadata that in some way is connected to the measurement of exposure level. All strategies in the presented datasets can be used for this purpose if the worker category (and not task) are used as metadata. This will give more crude estimates as all the workers do a variety of tasks. If the B strategy can be combined with activity data from other sources, for example, work logs or maintenance logs, collection of activity data might be available without much effort.

The low number of measurements in dataset A that have been labeled “some flushing” can explain that the difference in baseline of “much” flushing is not significant in the mixed model analysis. Other work not categorized as flushing is also exposed, and it seems like “some flushing” is less exposed than those not flushing. This can be caused by different work in different groups. For example, “flushing” is not used as a category among plant workers in dataset C, although they do flush during daily cleaning operations. This illustrates that categories can be given different meanings by the workers.

The estimated variance from the mixed model matrix on index values is dominated by the within worker variance. This supports the description of H_2_S as an exposure with unpredictable potential. In dataset C approximately 71% of the total variance was within worker, while in dataset A approximately 100%. The main part of the variance is due to the large number of different tasks.

Sampling strategy A has found both low and very high levels, showing the high variability in exposure. A higher variability will increase the estimate of the 95th percentile of exposure. Routine collection as in B seems to give some of the same variability, but because of the large number of measurements, the opportunity to use the data histogram directly to evaluate the percentiles are present, instead of calculating them statistically. The strategy in C will also give the same histogram opportunities, but depend on “luck” to get the highest exposures, as the measurements are made over selected periods of time and will not always contain the highest exposures. The 95th percentile estimate might nevertheless be the same in B and C, but the histogram in B is expected to show higher values within the values above the 95th percentile due to the collection being also based on alarms.

## Conclusions

Assessment strategies based on campaign measurements are not suitable for unpredictable exposures of agents with life-threatening potential, like H_2_S. For such exposure, the use of an index that includes peak exposure measures, gives a better foundation of risk perception than TWA.

To rely on experience of H_2_S exposure is not sufficient for workers protection, as the underlying risk of sudden high exposure is always present. Low TWA levels are not enough to document low-risk levels for exposure with acute potential. The presented data shows that TWA values of measurements with infrequent high peaks easily camouflage dangerous acute exposure levels. The use of continuous surveillance for risk warning should be standard procedure in work with exposure to chemical agents with immediate danger to life and health. Exposures with a ceiling limit should also be monitored this way.

Datasets B and C are systematically collected from personal electrochemical gas detector alarms with data logging used for risk warning. The study shows that such data can also be used and presented for exposure characterization. If possible, it should be combined with the collection of activity data. Occupational hygienists and occupational medicine personnel should take this method into account when evaluating collection methods and effects of exposure on workers.

Water distribution network seems to also have incidents of high exposure. This may be due to connections between water manholes and wastewater networks. No other explanation is indicated in the data.

## Supplementary Material

wxae043_suppl_Supplementary_Material

## Data Availability

Dataset B and C will be made available at Sikt – Norwegian Agency for Shared Services in Education and Research (www.SIKT.no), https://sikt.no/tjenester/finn-data/surveybanken, with “H_2_S” as searchable variable. Dataset A is available at https://doi.org/10.18712/NSD-NSD2964-V1. The algorithm is available online as a Supplementary File to Austigard et al. 2023. For the purpose of open access, the author has applied a CC BY public copyright licence to any Author Accepted Manuscript (AAM) version arising from this submission.
