# Supplementary material for: Comparison of 3 methods characterizing H2S exposure in water and wastewater management work
Source: Ann Work Expo Health. 2024 Jul 9;68(7):725–36. doi: 10.1093/annweh/wxae043 (PMC11306318; doi:10.1093/annweh/wxae043)
Supplement: wxae043_suppl_Supplementary_Material [file wxae043_suppl_supplementary_material.pdf]

Supplementary materials

To:

Comparison of three methods characterising H_2S exposure in water and wastewater management work

by

Åse Dalseth Austigard [1, 3] Hans Thore Smedbold [2,4] Kristin von Hirsch Svendsen [1]

[1] Department of Industrial Economics and Technology Management, NTNU - Norwegian University of Science and Technology, PO Box 8900, Torgarden, N-7491 Trondheim, Norway.

[2] Department of Occupational Medicine, St Olav University Hospital, PO Box 3250, Torgarden, N-7006 Trondheim, Norway

[3] Trondheim Municipality, Working Environment Office, PO. box 2300 Torgarden, N-7004 Trondheim, Norway. E-mail: ase-dalseth.austigard@trondheim.kommune.no Tel.: +47 95263902

[4] Department of Public Health and Nursing, Faculty of Medicine and Health Sciences, NTNU, PO Box 8900, Torgarden, N-7491 Trondheim, Norway

6 pages.

I:	Logbook form – translated	1 page
II:	Data description details dataset C	4 pages

Registration form of activity and alarms: New form each day!

Start day	Name		End day		
	Date				

Continuous registration through the day:

**Location/ place**	**Tasks**		**Start-time**	**Duration**	**Comments**
Car number<br>Facility number<br>Net system<br>Office<br>Restroom	Exposed tasks<br>1 Flushing<br>2 Sludge/ septic<br>3 Other cleaning<br>4 Repair/ maintanance<br>5 Inspection, no raise<br>6 Stearing of camera, above ground<br>7 Rigging up/ down*<br>8 In pit/ manhole<br>9 Other exposed (describe)	Unexposed tasks:<br>11 Driving<br>12 Monitor operator TV car<br>13 Meetings<br>14 Reports/ office<br>15 Breake<br>16 Waternet without sewage cont.<br>17 Control room<br><br>19 Other unexposed (describe)	(hh.mm)	minutes	For example:<br>Amount of water filled<br>Type of flushing hood<br>Amount emptied mass (m^3)<br>Kind of emptied mass (sand, septic, fat, …)<br>Other comments

* before flushing or other kinds of exposed tasks, after opening of manhole cover, or entering a facility, or recognising the smell of sewage.

II: Data description details dataset C

Of the 1807 registered days, we have context information for 1467 days: 977 from registered logbooks and 490 from other sources. Real zero exposure days were observed in 449 of the registered days. This includes 419 of the workdays with missing measurements. These 419 workdays, include one person who ended the employment during the period (20 days), and one person who started during the period (10 days). This gives us 872 days with measurement data. Seven of the days with registered measurements above LOD (three above CV) are from registered real zero days, indicating that the equipment has been used by other persons. These seven days are included in days with no context info. The number of days with missing data was 198 (11 %). Days with complete set of information, meaning both measurement and context present, or being identified as not exposed, account for 64 % of the workdays. The SEGs ;Wastewater net, Pumping station, Water net and Plant, have 73 %, 68 %, 65 % and 46 % respectively. Sample description data are shown in Table SII-1.

Table SII-1: Sample description. Total sample is 1807 person workdays. Seven real zero days with measurement data above LOD are included in data without context information.

		Measurement present (N=872 (48 %))			No measurement (N=935 (52 %))
		Above 10 ppm (N=118 (7 %))	LOD to 10 ppm (N=404 (22 %))	Under LOD (N=350 (19 %))	
Context info present	Possibly exposed	106 (6 %)	357 (20 %)	244 (14 %)	318 (18 %)
	Real zero	~~3 (0.2 %)~~	~~4 (0.2 %)~~	23 (1 %)	419 (23 %)
No context info		9+3 (1 %)	43+4 (2 %)	83 (5 %)	198 (11 %)
In measured days (N=872)		14 %	46	40	

The data collected are from days covering the range of normal weather for the area. Temperatures were between -13 and +26°C, and day mean from -8 to +20°C. Wind has been up to 12m/s, with day mean mostly under 5 m/s. Most rain was in the September periode, with day maximum 23mm/24h. All three sample periods had rainfall or snow (max snow equivalent to 15 mm rain/24 h). Data is from Voll Weather station in Trondheim, altitude 127 m. Downloaded from online archives of Norwegian Meteorological Institute (www.met.no).

Table SII-2: Selection of the 20 highest values on each parameter, in total 38 cases with the rankings. Sorted by max H2S-level up to place 20 (gray background color), then the same for TWA (+14 cases) and index (+4 cases). SEG codes are: 1: Water net; 2: Plant; 3: Pumping stations; 4: Wastewater net.

Measurement identification			Level			Place		
ID	SEG	LogUS_nr	TWA	Index	Max	TWA	Index	Max
52	1	1255	0,0748	59	28	1	1	1
22	1	1465	0,0174	35	22	9	9	2
1	2	1264	0,011	32	21	23	15	3
56	3	1079	0,011	32	21	24	14	4
59	3	1082	0,0669	47	20	2	2	5
40	4	40	0,0096	30	19	31	20	6
1	2	663	0,0092	29	18	32	21	7
1	2	603	0,0092	29	18	33	22	8
46	2	648	0,0126	35	17	18	10	9
35	1	1358	0,0089	28	17	34	23	10
1	2	783	0,0088	28	17	37	25	11
54	4	776	0,0135	30	17	14	19	12
33	4	333	0,0145	39	16	12	5	13
24	2	1467	0,0084	27	16	39	27	14
14	1	977	0,0099	28	15	29	26	15
1	2	1566	0,008	27	15	43	29	16
40	4	580	0,008	27	15	44	31	17
17	4	1100	0,008	27	15	45	30	18
14	1	1037	0,0079	26	15	47	32	19
1	2	1084	0,0079	26	15	48	33	20
28	3	1351	0,0303	41	13	3	3	57
28	3	1593	0,0245	27	12	4	28	78
59	3	1564	0,0235	28	12	5	24	82
57	2	177	0,0229	33	11	6	12	91
1	2	1324	0,0217	6	5	7	>100	>100
47	4	709	0,0207	16	7,4	8	>100	>100
24	2	1347	0,0166	26	12	10	35	79
48	2	1613	0,0159	41	12	11	4	77
38	4	278	0,0142	32	14	13	13	35
57	2	297	0,013	37	15	15	6	25
42	4	1667	0,0129	33	15	16	11	27
1	2	1	0,0128	36	14	17	7	40
54	4	1619	0,0123	25	13	19	48	65
40	4	1605	0,0123	25	13	20	49	66
31	3	91	0,012	35	13	21	8	67
17	4	1340	0,0116	32	14	22	16	30
47	4	107	0,0108	31	14	25	17	42
57	2	357	0,0104	31	13	28	18	54

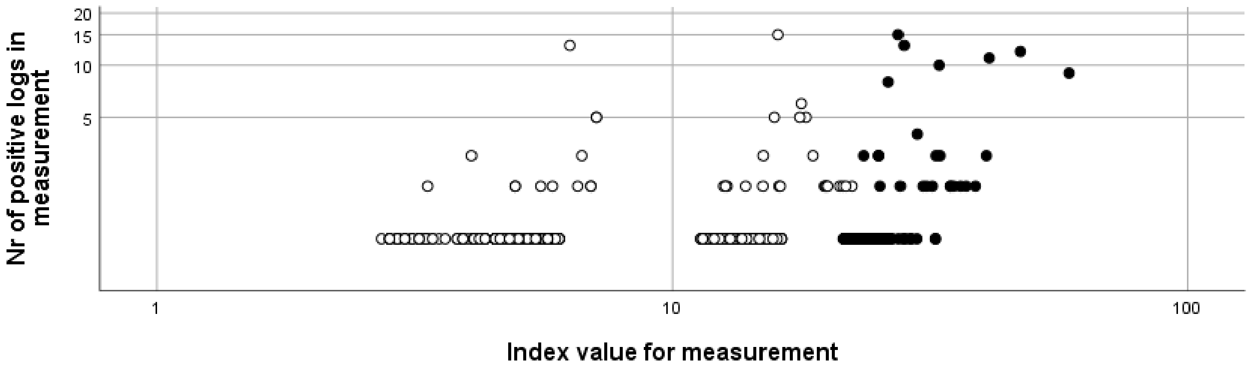

Figure SII-1: Plot of number of positive values against index value of measurement. Filled circles exceed ceiling value (CV).

Relative to the number of persons in the different SEGs, Water net workers have less weeks that are unexposed or below LOD than the other SEGs (41 % compared to 47-49 %). In Figure SII-2 we present the relative distribution of maximum number of days with detected exposure for each person each week (person week). The "zero" part is divided into "All days real zero" and "0 exposed days detected". Logs for these 60 persons in 6 weeks give a total of 360 person weeks. Seven percent of person weeks have exposure above LOD each of the five workdays of the week. These numbers include all days, also those without measurements, so with measurements on all exposed workdays, we should expect higher fractions on the right end of the scale and less on the lower end.

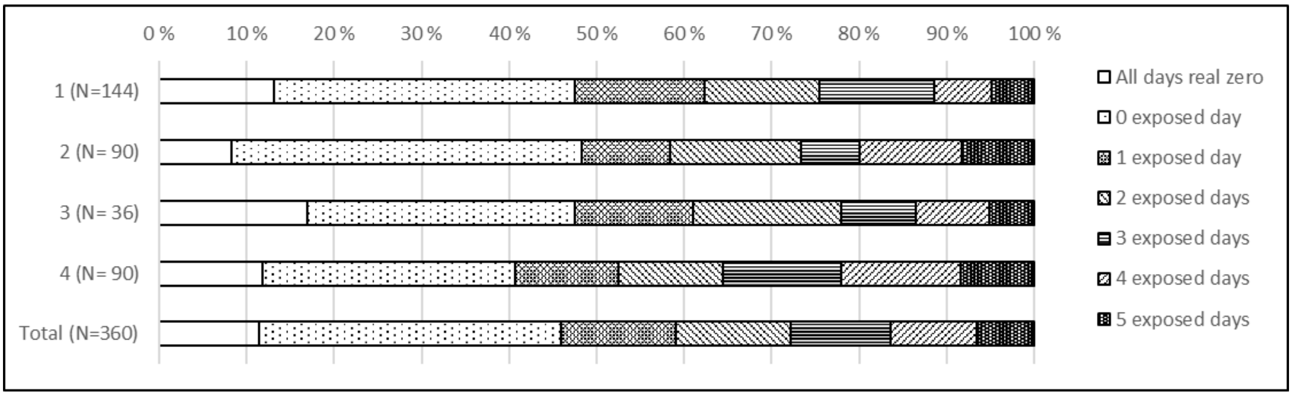

Figure SII-2: Relative distribution of number of days with detected exposure of H_2S above LOD (exposed days) in person week (60 persons, 6 weeks - this gives a total of 360 person weeks), presented per SEG and in total. The zero days-category is divided into 'All days true zero' and '0 exposed days above LOD'.

NB: Altered SEG number: SEG 1= Wastewater net, 2= Plant, 3= Pumping stations, 4= Water distribution net.

Distribution of accumulated measurements at different exposure levels for the different datasets. Figure SII-3 for all measurements, and Figure SII-4 for measurements above LOD.

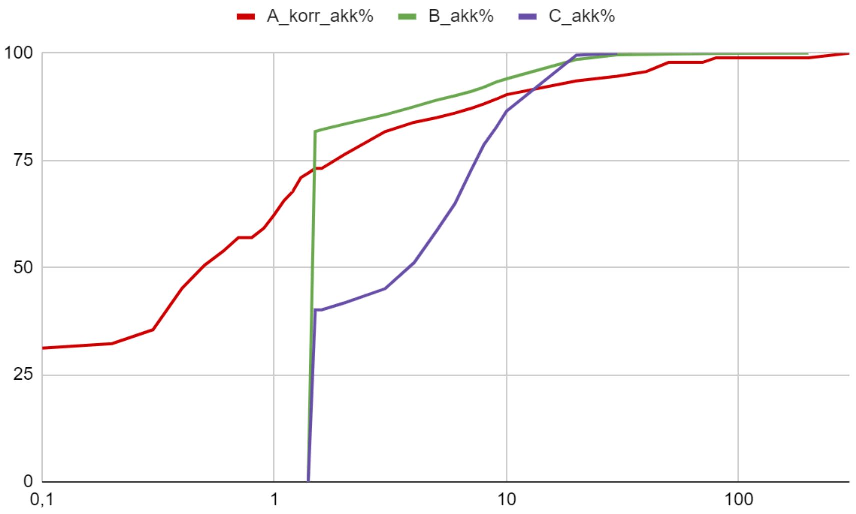

Figure SII-3: Distribution of max H_2S level in measurement, for all measurements for the different datasets.

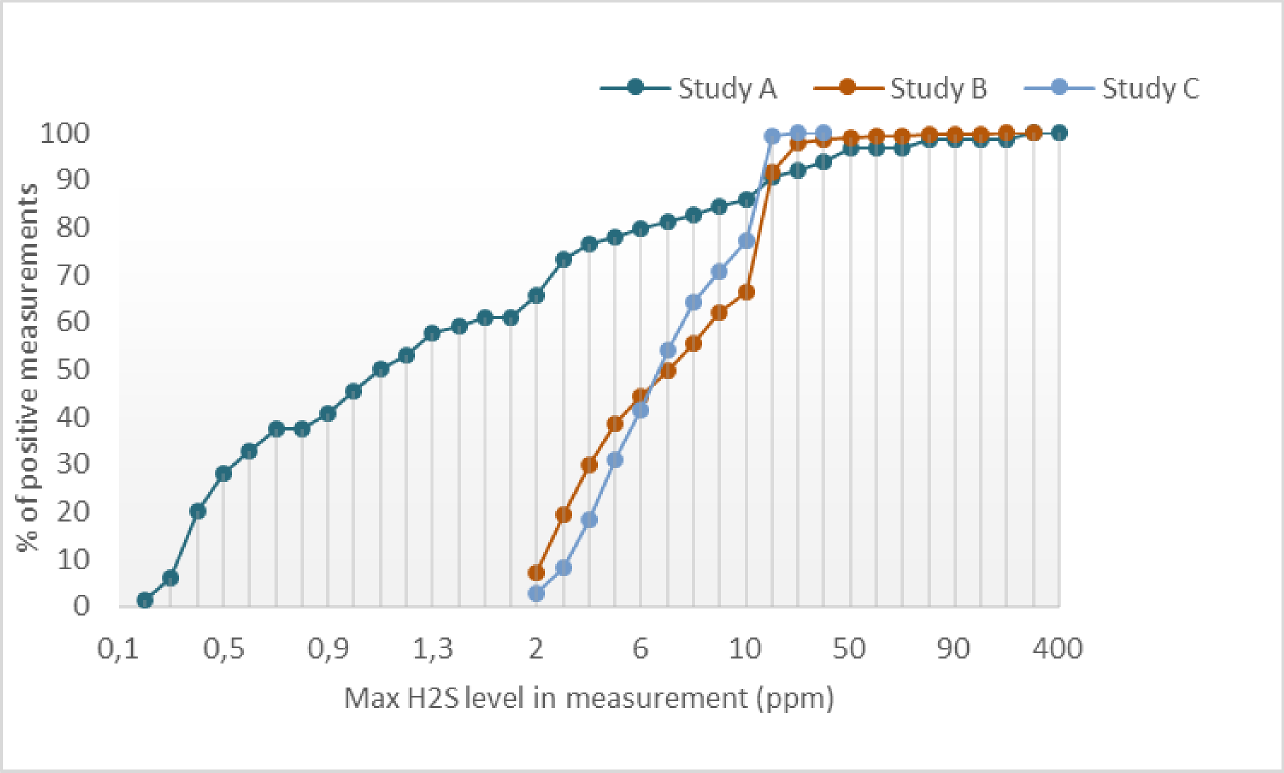

Figure SII-4: Distribution of max H_2S level in measurement, for measurements above LOD for the different datasets.
